# A Study of the Effects of Exercise on the Urinary Metabolome Using Normalisation to Individual Metabolic Output

**DOI:** 10.3390/metabo5010119

**Published:** 2015-02-27

**Authors:** Evangelia Daskalaki, Gavin Blackburn, Gabriela Kalna, Tong Zhang, Nahoum Anthony, David G. Watson

**Affiliations:** 1 Strathclyde Institute of Pharmacy and Biomedical Sciences, University of Strathclyde, Glasgow G4 0RE UK; E-Mails: evangelia.daskalaki@strath.ac.uk (E.D.); tong.zhang.101@strath.ac.uk (T.Z.); nahoum.anthony@strath.ac.uk (N.A.); 2 Glasgow Polyomics, University of Glasgow, Wolfson Wohl Cancer Research Centre, Glasgow G61 1 BD, UK; E-Mail: gavin.blackburn@glasgow.ac.uk; 3 The Beatson Institute for Cancer Research, Garscube Estate, Glasgow G61 1BD, UK; E-Mail: g.kalna@beatson.gla.ac.uk

**Keywords:** exercise, urine, metabolomics, normalisation, mass spectrometry

## Abstract

Aerobic exercise, in spite of its multi-organ benefit and potent effect on the metabolome, has yet to be investigated comprehensively via an untargeted metabolomics technology. We conducted an exploratory untargeted liquid chromatography mass spectrometry study to investigate the effects of a one-h aerobic exercise session in the urine of three physically active males. Individual urine samples were collected over a 37-h protocol (two pre-exercise and eight post-exercise). Raw data were subjected to a variety of normalization techniques, with the most effective measure dividing each metabolite by the sum response of that metabolite for each individual across the 37-h protocol expressed as a percentage. This allowed the metabolite responses to be plotted on a normalised scale. Our results highlight significant metabolites located in the following systems: purine pathway, tryptophan metabolism, carnitine metabolism, cortisol metabolism, androgen metabolism, amino acid oxidation, as well as metabolites from the gastrointestinal microbiome. Many of the significant changes observed in our pilot investigation mirror previous research studies, of various methodological designs, published within the last 15 years, although they have never been reported at the same time in a single study.

## 1. Introduction

From the smell and colour of urine as a tool for disease diagnosis [1] to the elusive promise of an “exercise pill” as a preventative measure of disease [2,3], could this be the future of health prescription? How could exercise, with its multi-factorial and multi-organ health benefits [2,4,5,6,7], be successfully administrated as an oral medication? If this theoretical pill existed, it would have to replicate the downstream metabolic effects of a single type of exercise tailored specifically to each unique metabolome or even metabotype [8,9]. Exercise, as an external challenge to the human metabolome, creates an immediate response (turn-over rate in seconds [10]) across this biological matrix, which, unlike studies investigating the effects of fasting, have been shown to exhibit large inter-subject variability [8,11]. Therefore, the question of dosing specific exercise regimes to varying population cohorts (sedentary *vs.* regularly active *vs.* athletes) in order to maximize the efficacy of the intervention remains to be answered. Therefore, how can we, by utilising evidence-based markers, predict the intensity and training modality that would ensure a quicker adaptation and improved outcome in these subgroups? The answer could lie within the plethora of human metabolism, which, in spite of its vast number of constituents, still is the most sensitive measure to investigate cellular and human phenotype [1,9,10]. 

As such, metabolomics, the rapidly developing omics technology, allows for hundreds of metabolites (generally with a mass <1500 Da, at ≤5 ppm mass deviation) to be investigated within each metabolome at any given time-frame, creating a ‘snapshot’ of the biological state of an organism [12]. A number of metabolomics-based studies have provided evidence to suggest that there is a clear effect of exercise on the human metabolome [4,13,14,15]. The most persistent observations concern effects on the purine pathway, highlighting increases in adenine nucleotides (AdN) in both acute and prolonged exercise regimes [16,17,18,19,20,21,22,23], with reduced excretion exhibited following an adaptative response [23] that is accommodated by a reduced level of resting hypoxanthine [17,22,24,25,26,27]. Moreover, Hellsten *et al.* [28] showed that muscle urate, as well as allantoin levels, the latter being a product of urate oxidation, were increased in habitually active male subjects following exhaustive exercise. Allantoin can only be formed non-enzymatically in humans, and it was concluded that uric acid was acting as an antioxidant against reactive oxygen species (ROS) generated during exercise. Hence, this could be a useful tool in examining levels of oxidative stress. As previously mentioned, plasma levels of hypoxanthine were also increased after exercise, and this may result from xanthine dehydrogenase being a rate limiting enzyme in urate formation [24]. In muscle, ATP is degraded to hypoxanthine, which is lost from the muscle, but may be salvaged by hypoxanthine-guanine phosphoribosyl transferase. Several papers have observed that hypoxanthine salvage tends to be more efficient in trained individuals and that, along with other purine metabolites, can provide an indication of the effectiveness of a training regime [26]. A targeted metabolomics study examining approximately 200 plasma metabolites in relation to exercise in individuals from a longitudinal cohort study concluded that metabolic profiles obtained during exercise gave a signature of exercise performance, as well as cardiovascular disease susceptibility [5]. Important marker metabolites for the effect of exercise included purine metabolites, tryptophan metabolites, citrulline (marker of nitric oxide formation and present to a lesser extent in the plasma of fitter individuals) and, finally, nicotinamide (tryptophan metabolite), which is known to enhance insulin release [5]. Lustgarten *et al.* [29] noted a positive correlation between maximum oxygen consumption ($\dot{V}$O_2max_) with tryptophan, and an increase in tryptophan-related metabolites, such as kynurenate, was exhibited in a study of individuals after running the 26.2-mile Boston marathon. A ^1^H-NMR investigation into same sex twins looked into the effect of prolonged physical activity adherence to metabolic and gene expression links [4]. Numerous differences were found between persistently physically active and inactive individuals in the circulating metabolome, and the results reflected better cardio-metabolic health in the physically active twin [4].

Apart from the aforementioned studies, there have been very few comprehensive liquid chromatography mass spectrometry (LC-MS)-based investigations utilising an untargeted metabolomics method. The research conducted by Lewis *et al.* [5] and the Nieman *et al.* [13,30] on the effects of exercise have set a standard for future work; however, there are new prospects available in metabolite coverage and understanding due to the rise of high resolution and high throughput monitoring systems. Therefore, we conducted an exploratory, hypothesis generating pilot study utilising an untargeted LC-MS method in order to explore the effects of a one-h aerobic exercise challenge in the urine metabolome of three physically active males. We utilised our well-established hydrophilic interaction chromatography (HILIC) method to carry out the analysis [30,31,32]. The use of urine provides an ideal non-invasive method that results in a large overview of the metabolite matrix. As serialised time points, each individual urine sample provides an averaged response of the metabolic output of a particular individual and, in doing so, provides a unique overview of the daily metabolite variation. Hence, we devised a continuous 37-h protocol with sampling pre- and post-exercise accounting for the majority of the time-points (across 31 h) in order to generate an understanding of the acute effects of exercise and, where possible, the duration of that response in the metabolite profiles. 

## 2. Materials and Methods

### 2.1. Ethics Statement (UEC 14/28, Watson/Daskalaki: Pilot Exercise Trial)

This study was approved by the Ethics Committee of the University of Strathclyde (Glasgow, UK) and conformed to the Declaration of Helsinki. Prior to successful recruitment, all subjects completed a physical activity readiness questionnaire, as well as a health questionnaire, in order to assess physical activity levels and to ensure that they had no history of the following: anaemia, diabetes, epilepsy, as well as any underlying respiratory or cardiovascular complications. All subjects gave written informed consent to participate.

### 2.2. Subjects and Experimental Design

**Figure 1:**
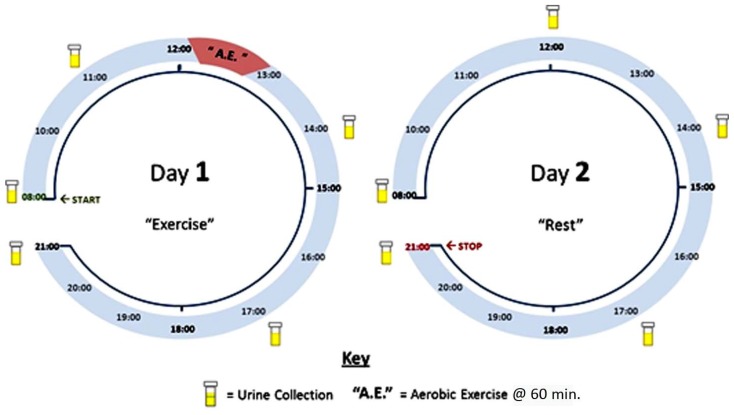
Illustration of experimental design and sample collection.

Three physically active, non-smoking males (age range: 32–38 years) participated in the pilot study. The subjects were regularly engaged in predominantly running, long-distance walking and cycling. For at least two weeks prior to study commencement, subjects were asked to abstain from taking any sport or nutritional supplements and had no sign of illness. Subjects underwent a two-day (37 hour) trial, whereby urine samples were collected at regular intervals across this timeline: Day 1, ~08:00 Pre-exercise (P) 1 , first pass urine), 11:00 (P2), 14:00 (Post-exercise (PT) 1 17:00 (PT2) and 21:00 (PT3); and Day 2, ~8:00 (PT4; first pass urine), 12:00 (PT5), 14:00 (PT6), 17:00 (PT7) and 21:00 (PT8)). Aerobic exercise was performed at the Strathclyde Sport and Recreational Centre (Glasgow, U.K.) on Day 1 between 12:00 and 13:00 (referred to as the A.E. session in this diagram) on either a treadmill, bicycle ergometer or in a combination. Subjects were permitted to control their own pace and drink water *ad libitum*, but had to be engaged in activity for at least 50 min. After the exercise session and for the remainder of the sample collection timeline, subjects were asked to refrain from any physical activity. Figure 1 provides a full illustration of the two-day protocol.

### 2.3. Sample Collection and Preparation 

Coded, pre-labelled sterilised urine containers were distributed to the subjects along with cool bags (Sistema Plastics, available from Amazon Co., Slough, UK) for storage and transport to the laboratory located at the Strathclyde Institute of Pharmacy and Biomedical Sciences. A designated drop-off section (at a temperature of −35 °C) was created for the samples. The unopened urine containers were stored for a maximum of two weeks prior to being thawed at room temperature and prepared for LC-MS analysis. For the analysis , using a ZIC-pHILIC column, 200 μL of urine were thoroughly mixed with 800 μL of ACN, followed by centrifugation at 7840 relative centrifugal force for 5 min; 800 μL of supernatant were then transferred to an LC auto-sampler vial (Thermo Fisher, UK). For quality control purposes, a pooled sample representing all subjects, as well as a subject-specific pooled sample were prepared (the latter utilised as a normalisation technique). For the pooled samples, 100 μL of urine were gathered from each sample and then treated as above. Four standard mixtures containing 150 authentic standards were run also after the samples.

### 2.4. Measurement of Creatinine 

Fifty microlitres of diluted samples and prepared creatinine standard stock solutions were thoroughly mixed with 100 μL of creatinine detection reagent (Enzo Life Sciences, Exeter, UK) in a 96-well plate. Absorbance was read at 490 nm via a Spectra Max M5 from Molecular Devices. The concentrations of creatinine in the test samples were calculated as stated previously in the literature [31].

### 2.5. Chemicals and Solvents

HPLC-grade acetonitrile (ACN) was purchased from Fisher Scientific, U.K., and HPLC-grade water was produced by a Direct-Q 3 Ultrapure Water System (Millipore, U.K.). AnalaR-grade formic acid (98%) was obtained from BDH-Merck (Poole, Dorset, U.K.). Authentic stock standards were prepared as stated previously in the literature [30] and diluted 4 times with ACN before LC-MS analysis. Ammonium carbonate was purchased from Sigma-Aldrich, U.K.

### 2.6. LC-MS Method

LC-MS data were acquired on an Accela HPLC (Thermo Fisher Scientific) coupled to an Exactive Orbitrap (Thermo Fisher Scientific, Hemel Hempstead, UK) in both positive and negative mode set at 50,000 resolution (controlled by Xcalibur version 2.1.0; Thermo Fisher Corporation, Hemel Hempstead, UK). The mass scanning range was *m/z* 75–1200; the capillary temperature was 320 °C; and the sheath and auxiliary gas flow rates were 50 and 17 arbitrary units, respectively. The separation was performed on a ZIC-pHILIC column (150 × 4.6 mm, 5 μm from HiChrom, Reading, UK) in binary gradient mode. The mobile phase used was 20 mM ammonium carbonate buffer (pH 9.2) and pure ACN; the flow rate was 300 μL·min^−1^. The gradient was programed as follows: 0 min 20% A/80% B to time 30 min 80% A/20% B. The injection volume was 10 μL, and the sample tray temperature was controlled at 12 °C during the measurement. Samples were run in a stratified method with between-subject samples placed in randomised order.

### 2.7. LC-MS Data Processing with MzMatch and Ideom (Version 19)

Raw LC-MS files were converted to mzXML (ProteoWizard) and separated into ESI positive and negative. Converted files were then processed with open source MzMatch (http://mzmatch.sourceforge.net/) and the identification of putative metabolites was made via the macro-enabled Excel file, Ideom (http://mzmatch.sourceforge.net/ideom.html). Details regarding the data processing, metabolite identification, as well as databases available through Ideom can be found in previous literature [33]. Details of the R script for data processing with MzMatch and Ideom configurations can be found in supplementary File S1, R script, and Table S1, Ideom settings, respectively.

### 2.8. Statistical Analysis 

Graphical representations, tabular features and statistical analysis (*p*-value generation) were performed in Excel (Microsoft Office 2013). Raw data were subjected to a variety of normalisation techniques; pooled subject-specific MS creatinine samples, MS and spectrophotometric creatinine, as well as a subject-specific area percentage. For the latter, the first step includes calculating the sum of the peak areas of each metabolite across the 37-h protocol for each subject individually. Secondly, each respective metabolite response from every time point is then divided by the subject specific sum and multiplied by 100. Paired *t*-tests and fold changes were calculated on the new area percentage dataset. In order to observe the effect of the various normalisation strategies, data were also subjected to unsupervised PCA (scaled to unit variance) via SIMCA-P 13 (Umetrics, Sweden). 

## 3. Results 

### Normalisation

**Figure 2:**
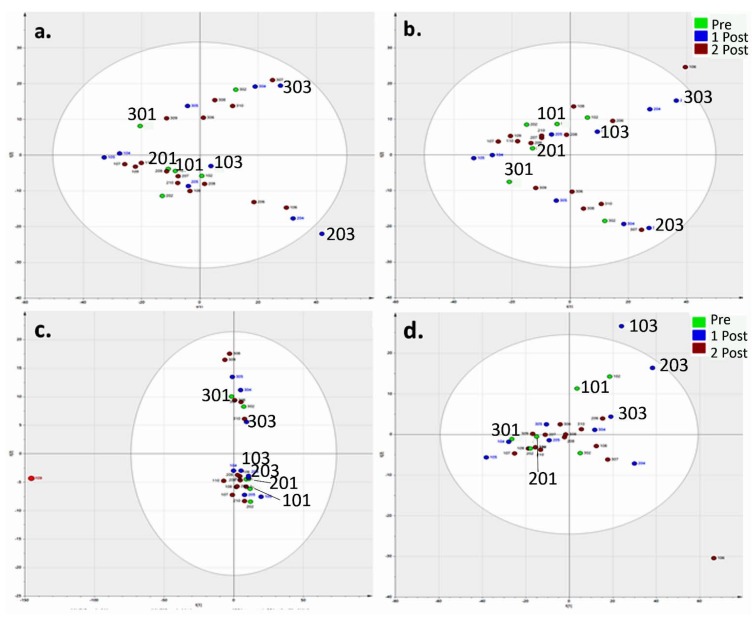
Normalisation strategies applied to the urine metabolome of three subjects (~1,000 putative metabolites). PCA of data normalised to: (**a**) the MS creatinine value of each unique sample detected exhibiting a similar pattern to raw data (R2X: 0.721; Q2: 0.432); (**b**) the MS creatinine value of subject-specific pooled samples (*i.e.*, pooled Subject 1 creatinine value utilised to normalise all of Subject 1’s raw data, and so on; R2X: 0.767; Q2: 0.489); (**c**) spectrophotometric creatinine value (the normalised data are closely clustered in the PCA model with the exception of all sampling time-points from Subject 3; R2X: 0.883; Q2: 0.468); and (**d**) subject-specific area percentage (R2X: 0.704; Q2: 0.447; showing the smallest difference between the goodness of fit and predicted fit). The PRE group includes Pre 1 (P1) and P2; the POST1 group includes Post 1 (PT1), 2 and 3; POST2 includes PT4, 5, 6, 7 and 8.

Since the strength of urine can vary, a major question in urinary metabolomics is how does one normalise to allow for variation in strength? Creatinine normalisation is often used, yet the reliability of such a method is uncertain; this is, in fact, discussed at length in the review on urinary normalization techniques by Ryan *et al.* [34]. It is clear from a number of metabolomics studies examining differences between individuals that each person presents a unique metabolic profile [8,11]. These inter-individual variations can be seen between the clustering patterns of the principal component analysis (PCA), where data have been normalised to either: (1) MS creatinine; (2) pooled subject-specific MS creatinine; or (3) spectrophotometric creatinine (Figure 2). The result of normalising to MS response for creatinine (Figure 2a) does not vary from the original raw dataset and does not improve the observed clustering between the subjects. However, when attempting to normalise to pooled subject-specific (Figure 2b) and spectrophotometric (Figure 2c) creatinine, the results vary considerably, especially for the latter. Having previously seen the clustering of each subject uniquely, the results of normalisation via the assay kit aid in the potential further differentiation of two metabotypes within our results. In Figure 2c, all of Subject 3’s samples are separated from Subjects 1 and 2. Given that all our subjects were healthy, non-smoking and regularly active, this could be an additional factor in determining relative fitness prior to any form of exercise challenge due to the relationship of creatinine clearance and muscle activity. The most effective strategy, however, can be seen in Figure 1d, as the data are normalised to the subject-specific area percentage. This approach has not been widely used, but there is some justification for doing this, as an almost complete recording for each metabolite was collected over the designated time-frame of the study. There are very few studies that have collected urine samples separately over long periods, so there is no information variability of individual metabolites with time. Table 1 shows a range of metabolites in urine with their relative standard deviation (RSD) over 30 samples taken from our three subjects. Many of the amino acids have only small variations over time, indicating that they are sufficiently abundant in the body. However, this may suggest that they are not affected to such a great extent by this particular form of exercise; ultimately, maintaining a relatively constant output. On the other hand, there are some metabolites shown in Table 1 that are very variable over time. 

**Table 1:** RSD values for metabolites across 30 urine samples collected over 37 hours.

Compound	Mass	Retention Time (RT) (min)	RSD (%)
* Creatinine	113.050	10.1	16.3
* Threonine	119.058	14.9	25.4
Imidazolone propanoate	156.053	11.5	25.5
* Glutamine	146.069	15.5	27.2
* Serine	105.043	16.1	27.7
Dihydrothymine	128.058	15.2	31.2
N-acetylhistidine	197.080	10.6	31.5
Dimethylarginine	202.143	22.0	31.9
Dihydrouridine	246.085	10.8	32.0
* Betaine	117.079	11.7	32.3
* N-acetylglucosamine	221.090	12.2	33.2
N-acetylarginine	216.122	15.3	34.9
* N6-acetyllysine	188.116	15.5	35.6
* Adenine	135.055	9.5	37.9
* Methylthioadenosine	297.089	7.0	38.1
* Citrulline	175.096	15.9	39.0
* Proline	115.063	13.2	40.0
Methylimidazole acetic acid	140.058	9.7	41.0
* Alanine	89.048	15.2	41.3
* Methylhistidine	169.085	13.3	41.9
Thymine	126.043	12.0	42.3
* Isoleucine	131.095	11.6	42.5
Butenyl carnitine	229.131	9.8	42.8
Methylcytosine	125.059	11.0	43.2
* N-acetylglutamine	188.080	11.0	44.2
* Adenosine	267.097	9.3	44.9
* Phenylalanine	165.079	10.5	46.8
* Histidine	155.069	15.0	47.9
* Kynurenine	208.085	11.2	51.2
* Cytosine	111.043	11.6	51.1
* Ornithine	132.090	22.3	56.6
* Tryptophan	204.090	12.0	55.6
Carnitine	161.105	13.8	60.4
* Arginine	174.112	26.4	62.6
Creatinine (assay kit)	-	-	73.0
* Pantothenate	219.111	8.9	82.4
Tetrahydro aldosterone glucuronide	542.273	7.4	84.8
* Lysine	146.105	25.0	100.6
* Pyruvate	88.016	8.3	106.3
Cresol glucuronide	284.090	7.8	113.1
* Urate	168.028	13.0	121.6
* Xanthosine	284.075	12.7	141.3
* Tyrosine	181.074	13.3	133.7
* Inosine	268.081	11.2	180.3
* Hypoxanthine	136.038	10.5	186.1
Deoxyinosine	252.086	9.0	210.6

* Matches the retention time of an authentic standard.

**Figure 3:**
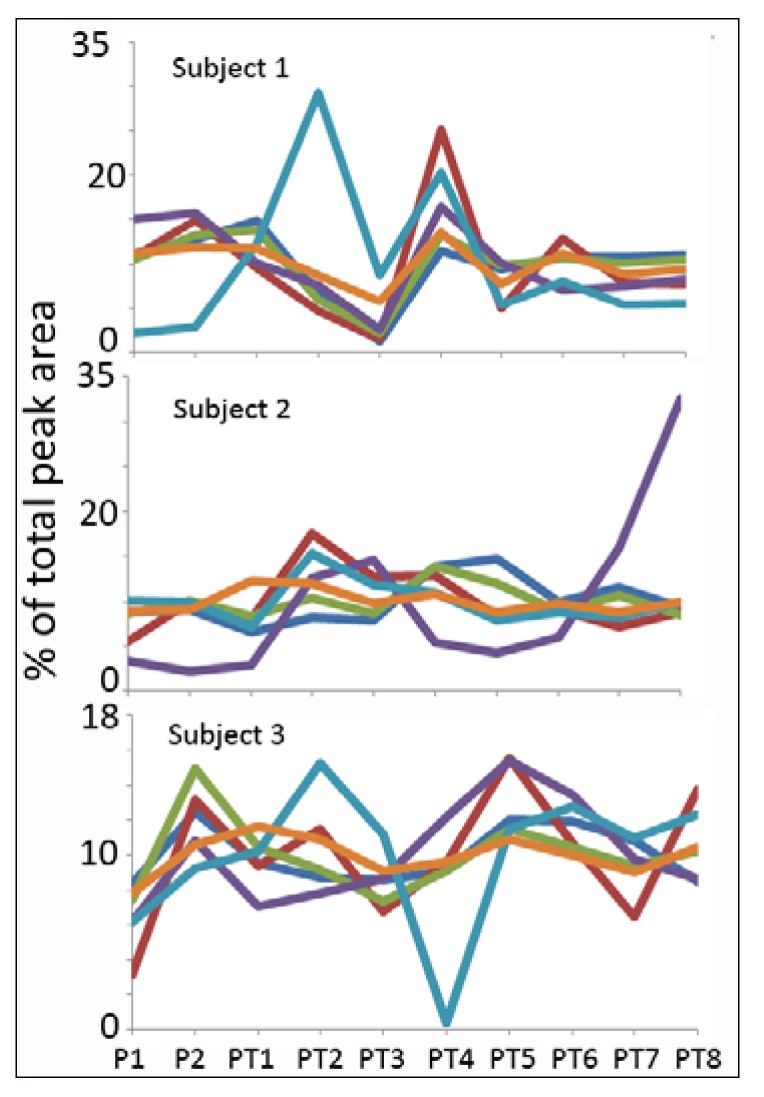
Comparison of the exercise response of the area percentage for MS creatinine (orange) with phenylalanine (red), carnitine (purple), threonine (green), glutamine (dark blue) and the dietary xenobiotic stachydrine (light blue).

**Figure 4:**
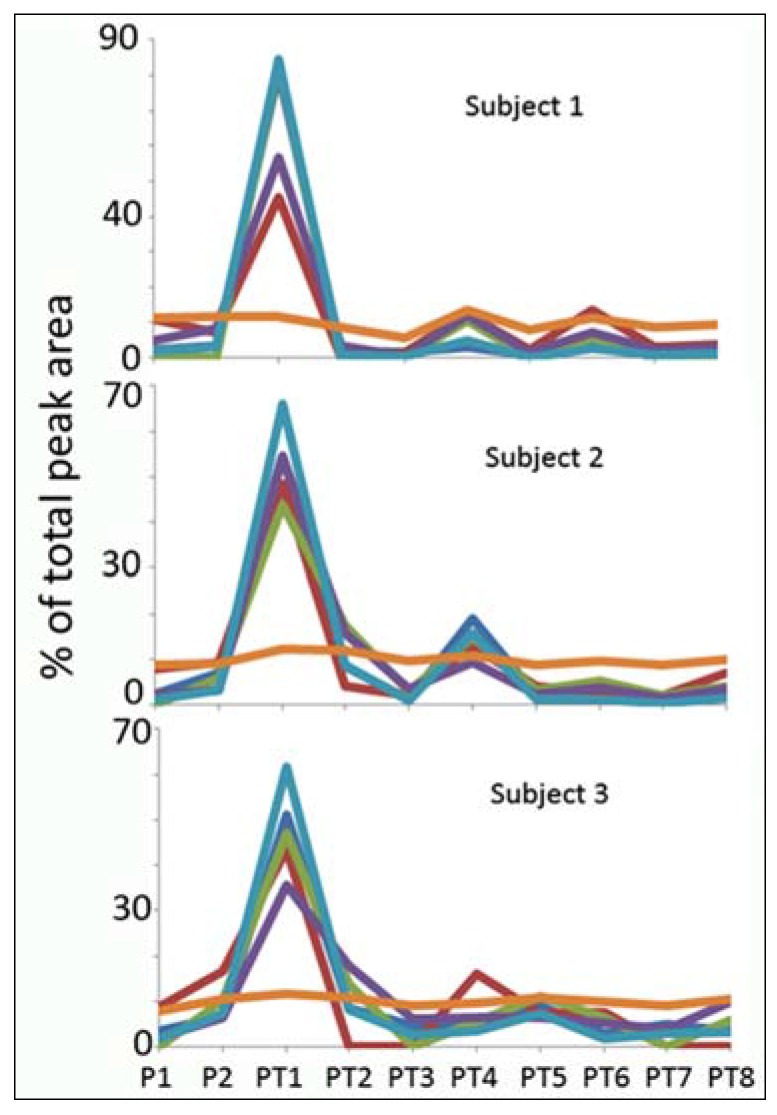
Comparison of the exercise response of the area percentage for MS creatinine (orange) with the levels of some metabolites from the purine pathway, hypoxanthine (light blue), xanthosine (green), inosine (red) and guanine (purple).

Figure 3 shows the variation in MS creatinine compared to some other metabolites (phenylalanine, carnitine, threonine, glutamine and stachydrine) that do not change very much over time for the three subjects; whereas, in Figure 4, variations in metabolites of the purine pathway (hypoxanthine, xanthosine, inosine and guanine) are shown in comparison to MS creatinine. As exhibited in Table 1, there are large variations across variable, as well as non-variable metabolites, and Figure 3 further emphasises that creatinine does not, in fact, follow a similar pattern to other metabolite markers. It is obvious from the similar trend response across the three subjects that the purines reflect a very strong acute impact of exercise; with peak levels observed at the first post-exercise sample (PT1). They also exhibit fluctuations over a smaller range on the following day. These metabolites are all in the pathway for ATP catabolism. The levels of adenosine (RSD in Table 1) were not affected to the same extent by exercise as the other purines, and thus, the breakdown of ATP appears not to proceed via this branch of the purine metabolism pathway. The effects of exercise and purine catabolism have been extensively described in the literature [20,21,22,23,24,25,26,27,28], and in particular, hypoxanthine has been the most studied of the purines.

Figure 4 shows the effects of exercise on four metabolites in the purine pathway in the three subjects. The four purines, hypoxanthine, inosine, xanthosine and guanine, fluctuate in a very similar manner and are all high in PT1; they also exhibit fluctuation over a smaller range on the following day. These metabolites are all in the pathway for ATP catabolism. The levels of adenosine (RSD in Table 1) were not affected to the same extent by exercise as the other purines, and thus, the breakdown of ATP appears not to proceed via this branch of the purine metabolism pathway. Effects on purine catabolism have been extensively described in the literature [20,21,22,23,24,25,26,27,28].

The possible permutations for comparison of the ten sampling points are large, so in order to investigate the impact of exercise on the wider metabolome, the first post-exercise sample was taken as the best to reflect the impact of exercise according to the previously established effect on purine metabolism. Hence, statistical analysis was carried out with the first post-exercise sample as the reference point in order to observe the metabolites that were most immediately impacted by exercise (Table 2). This would additionally provide a possible time series analysis in order to determine how long it took for the effect of the exercise intervention to subside. This adds statistical power, despite the small sample size, since nine points for each person can be referenced to the first post-exercise sample (PT1). Table 2 summarises the metabolites that were most significantly changed between the P1 and PT1 points. Having established the most significant changes between these two points, subsequent differences between PT1 and some of the other post-exercise points (PT2, 4, 5 and 7) were added to the table. Some of the metabolites affected by exercise took over 24 h to return to the level of the first pre-point. Comparison with the second pre-point (P2) did not produce as distinct differences for certain metabolites as can be seen in the comparison between PT1 and P1. This is probably due to the fact that the physical activity of the subjects (mainly in the morning travel methods of the subjects) prior to the exercise session was not strictly controlled. However, in many cases, there were still significant differences between P2 and PT1.

**Table 2:** Normalised urinary metabolites which are significantly changed by exercise for three subjects. P-value and ratio change for comparisons PT1 (first post-exercise sample) *vs.* P1 (pre 1), P2 (pre 2), PT4 (post 4), PT5 (post 6), PT5 (post 7) and PT7 (post 7). The majority of the metabolites match metabolites in the human metabolome to within 2 ppm and thus are characterised to MSI level 2 where alternative metabolites could be isomers of the identity assigned.

	Mass	RT (min)	PT1/P1		PT1/P2		PT1/PT2		PT1/PT4		PT1/PT5		PT1/PT7	
			Ratio	*p*-Value	Ratio	*p*-Value	Ratio	*p*-Value	Ratio	*p*-Value	Ratio	*p*-Value	Ratio	*p*-Value
Purine metabolism														
N2-N2-Dimethylguanosine	311.123	9.0	2.59	0.0200	1.57	0.11	1.97	0.12	1.18	0.61	2.68	0.070	4.75	0.0043
Xanthosine *	284.075	12.7	16.18	0.0191	8.24	0.019	3.98	0.014	5.25	0.022	13.7	0.017	18.6	0.019
Inosine *	268.081	11.2	581	0.045	10.8	0.048	5.55	0.049	5.64	0.0591	12.14	0.045	88.8	0.046
Deoxyinosine	252.086	9.0	43.3	0.010	15.6	0.0091	11.9	0.0060	8.87	0.0037	25.13	0.0062	53.4	0.0093
Guanine *	151.049	12.7	5.01	<0.001	4.17	0.0017	33.7	<0.001	3.30	<0.001	10.17	<0.001	27.7	<0.001
Hypoxanthine *	136.038	10.5	22.8	0.025	11.0	0.026	9.86	0.023	6.96	0.016	15.5	0.020	27.3	0.023
Kynurenine pathway														
** 3-Hydroxytryptophan	220.085	10.3	2.69	0.012	1.45	0.12	1.52	0.27	0.93	0.83	2.14	0.095	3.66	0.0095
^†^ Xanthurenic acid isomer	205.037	11.3	4.55	0.0023	1.93	0.0072	1.95	0.085	1.76	0.15	2.14	0.044	3.91	0.0001
Kynurenate	189.043	6.6	3.36	0.085	2.71	0.13	1.57	0.42	1.59	0.31	2.55	0.13	3.87	0.092
Hydroxytryptophol	177.079	7.5	3.74	0.0048	0.98	0.97	1.86	0.14	2.22	0.016	2.43	0.026	4.01	0.0040
N1-Methyl-2-pyridone-5-carboxamide	152.059	7.8	2.27	0.042	1.56	0.40	1.28	0.58	0.86	0.68	1.44	0.44	3.12	0.0012
Glycolysis														
Pyruvate *	88.016	8.3	6.79	0.026	5.93	0.02	2.11	0.12	2.81	0.041	9.08	0.024	6.50	0.031
Methyl oxalate	118.027	8.5	3.09	0.011	1.68	0.31	0.95	0.92	1.14	0.82	1.96	0.13	3.12	0.0026
Vitamins														
Riboflavin *	376.138	8.9	3.01	0.047	1.19	0.59	2.63	0.055	0.75	0.50	2.92	0.043	8.94	<0.001
Pantothenate *	219.111	8.9	5.09	0.031	3.18	0.048	1.96	0.13	2.19	0.071	3.44	0.034	4.37	0.042
Neurotransmitter metabolism														
L-Metanephrine *	197.105	18.6	4.56	0.013	2.00	0.050	1.88	0.13	1.45	0.42	2.90	0.035	5.21	0.026
Microbial metabolism														
Indole-3-acetyl-glutamine	303.122	8.2	4.86	0.010	1.61	0.26	3.36	0.024	1.38	0.28	3.64	0.021	8.02	0.011
5-Hydroxyindolepyruvate	219.053	5.3	3.99	0.0045	1.90	0.036	1.92	0.13	1.30	0.46	2.54	0.082	3.35	0.0067
Indoxyl sulphate	213.010	7.4	3.07	0.050	2.05	0.19	2.23	0.1155	1.48	0.4326	3.62	0.044	11.57	0.040
Hydroxyferulate	210.053	6.8	3.81	0.042	2.83	0.084	1.36	0.5378	1.55	0.27	6.27	0.040	5.87	0.044
Cresol sulphate	187.008	4.5	2.97	0.037	1.37	0.34	2.14	0.13	0.96	0.91	3.48	0.029	4.05	0.026
Phenol sulphate	173.999	5.0	3.32	0.0042	2.02	0.17	1.84	0.15	1.01	0.99	2.13	0.075	3.83	0.0034
Urocanate	138.043	7.5	2.81	0.054	2.03	0.036	1.73	0.16	2.02	0.049	2.97	0.027	5.50	0.014
Amino acids														
Tryptophan *	204.090	12.0	2.02	0.044	0.90	0.62	1.24	0.58	0.77	0.60	1.49	0.32	2.36	<0.001
O-Acetyl-L-homoserine	161.069	8.6	3.76	0.019	2.91	0.047	1.54	0.42	1.75	0.1177	3.06	0.023	4.23	0.019
Histidine *	155.069	15.0	0.41	0.047	0.39	0.073	0.67	0.44	0.62	0.30	0.29	<0.001	0.34	0.019
D-Methionine *	149.051	7.6	2.40	0.017	1.32	0.16	1.51	0.27	1.11	0.70	1.78	0.21	1.98	0.015
L-Proline *	115.063	13.2	3.88	0.018	1.85	0.091	2.18	0.058	1.74	0.12	2.38	0.047	3.53	0.035
Amino acid metabolism														
N-Acetylvanilalanine	253.095	8.0	7.48	0.0020	3.19	0.035	2.28	0.15	2.52	0.085	4.18	0.018	7.21	< 0.001
N-(Carboxyethyl) arginine	246.133	14.6	5.16	0.027	3.43	0.039	2.98	0.031	3.10	0.027	4.27	0.026	4.13	0.032
N-Acetyl-D-tryptophan	246.101	6.6	3.60	0.051	3.28	0.047	1.29	0.68	1.45	0.25	2.47	0.087	5.42	0.055
N-acetylmethionine	191.062	6.1	4.85	0.039	3.26	0.052	1.85	0.22	1.68	0.19	2.9	0.070	6.07	0.046
Amino acid oxidation														
Indole pyruvate	203.059	6.9	2.36	0.030	1.59	0.40	1.14	0.81	1.44	0.46	3.07	0.025	3.39	0.014
Acetamido-oxohexanoate	187.084	8.4	2.71	0.029	1.84	0.24	1.37	0.38	1.44	0.31	2.28	0.031	4.42	0.0075
Hydroxyphenylpyruvate	180.042	7.9	4.43	0.0030	2.63	0.077	1.54	0.41	1.80	0.12	4.66	0.0032	4.94	0.0051
Oxoarginine	173.080	14.7	1.99	0.04	1.78	0.089	1.96	0.052	1.63	0.078	2.88	0.018	2.18	0.045
Guanidovaleramide	158.117	24.7	6.74	0.0015	2.33	0.032	1.61	0.37	1.33	0.65	3.42	0.080	9.27	0.0049
Acetohydroxybutanoate	146.058	7.0	5.67	0.0016	3.51	0.0310	1.99	0.14	1.49	0.31	3.01	0.012	6.47	<0.001
Acetamidobutanoate	145.074	7.1	3.17	0.0095	2.24	0.14	1.69	0.28	1.80	0.13	2.82	0.029	6.39	0.0069
Amino acid oxidation														
Dihydroxymethylbutanoate	134.058	9.2	8.00	0.030	3.23	0.053	1.74	0.32	0.79	0.74	1.31	0.58	2.54	0.10
Methyl-oxopentanoic acid	130.063	5.2	4.21	0.026	2.65	0.068	1.51	0.45	1.18	0.66	2.32	0.084	3.68	0.030
Dioxopentanoate	130.027	8.8	2.31	0.046	1.56	0.39	1.10	0.85	0.94	0.84	1.47	0.31	2.18	0.035
Hydroxypentanoate	118.063	7.1	2.72	0.018	1.72	0.28	0.94	0.92	0.71	0.58	1.42	0.48	2.99	0.0035
Methyloxobutanoic acid	116.047	6.9	3.12	0.013	1.95	0.19	1.25	0.70	0.85	0.78	1.46	0.48	3.60	0.0020
Hydroxybutanoic acid	104.047	8.2	3.24	0.010	2.09	0.15	1.31	0.60	0.86	0.75	1.51	0.40	2.96	0.011
Carnitine metabolism														
Dodecenoylcarnitine	341.256	5.5	4.59	0.0025	3.00	0.030	2.67	0.045	1.52	0.13	3.55	0.051	5.19	< 0.001
Undecanoylcarnitine	329.256	5.5	3.08	0.026	1.57	0.20	1.48	0.35	1.32	0.40	2.07	0.17	3.83	0.028
Decanoylcarnitine	315.241	5.8	3.07	0.045	2.00	0.065	1.71	0.24	1.18	0.66	2.40	0.1425	5.05	<0.001
L-Octanoylcarnitine	287.209	6.4	2.15	0.016	1.41	0.19	1.52	0.31	0.71	0.51	1.46	0.47	3.18	0.004
Methylglutarylcarnitine	261.121	5.6	1.92	0.078	2.08	0.18	1.24	0.58	0.60	0.23	2.10	0.023	2.06	0.013
Hexanoylcarnitine	259.178	7.7	2.26	0.041	1.17	0.55	1.15	0.77	0.85	0.71	1.55	0.39	3.37	0.027
Valerylcarnitine	245.162	10.7	2.33	0.0186	1.74	0.065	1.36	0.42	0.98	0.96	2.52	0.0082	2.91	0.0075
Dehydroxycarnitine	145.110	15.8	2.76	0.0095	1.89	0.023	1.65	0.20	1.52	0.15	2.69	0.012	3.00	0.0082
Steroid metabolism														
Urocortisol glucuronide	542.273	7.4	6.48	0.0036	2.68	0.062	1.84	0.16	2.16	0.063	2.60	0.045	5.84	0.011
Dihydrocortisone glucuronide	540.257	5.5	5.47	0.029	2.59	0.082	3.70	0.028	2.04	0.075	3.65	0.032	8.43	0.033
Hydrocortisone sulphate	442.166	4.3	8.83	0.060	3.13	0.084	5.16	0.060	2.74	0.092	5.40	0.051	25.26	0.055
Hydroxyandrosterone glucuronide	482.252	5.5	4.27	0.0064	2.21	0.14	2.24	0.068	1.79	0.091	2.90	0.018	5.08	0.017
Androstane diol glucuronide	468.272	5.6	2.77	0.0082	2.11	0.17	1.71	0.21	1.24	0.51	2.38	0.065	4.45	0.0029
Androsterone glucuronide	466.257	4.8	3.59	0.0039	1.99	0.19	1.84	0.19	1.40	0.32	2.43	0.076	5.00	0.0069
Oxoandrostane glucuronide	480.236	5.3	4.13	0.046	2.17	0.15	2.03	0.13	1.47	0.32	2.83	0.061	9.22	0.051

* Metabolomics Standard Initiative Level 1: finding matches the retention time of the authentic standard; ** retention time earlier than the 5-hydroxytryptophan standard; ^†^ retention time later than the xanthurenic acid standard.

## 4. Discussion

### 4.1. Tryptophan Metabolism

In addition to the purine pathway, a number of other metabolite pathways were significantly affected by exercise. Lewis *et al.* [5] observed effects on the kynurenine pathway in response to exercise, and we observe the same type of effect in the current study with a weak effect on kynurenate and a clearer effect on 3-hydroxy tryptophan and hydroxyindole pyruvate, hydroxytryptophol, as well as pyridone carboxamide, which are also present in this pathway. Initially, we thought that xanthurenate (3-hydroxykynurenate) was also affected by exercise, but the affected compound shown in Table 2 appears to be an isomer of xanthurenate. Effects on tryptophan metabolism following exercise were also observed by Lustgarten *et al.* [29]. There is an early report that kynurenate can be converted non-enzymatically into 6-hydroxykynurenate, and this may occur in urine when levels of kynurenate are high [35]. We have previously observed in the completely different metabolic system of *Drosophila* that inhibition of the purine metabolism pathway with allopurinol caused a fall in metabolism within the kynurenine pathway [36]. 

### 4.2. Glycolysis

Pyruvate is strongly elevated by exercise, and this would be expected as a result of increased reliance on glycolysis as the source of energy with the decreased entry of pyruvate into the Krebs cycle. Lewis *et al.* [5] observed increased levels of pantothenate in plasma taken from marathon runners, and we also observe a large increase. It was proposed that elevated pantothenate resulted from an increased demand for CoA biosynthesis [5], but in the current case, pantothenic acid is excreted, which might reflect a decreased demand for CoA. This could also reflect a switch to glycolysis in place of fat metabolism. It has been observed that fat metabolism decreases with an increasing exercise intensity, and there is an increase in reliance on glycogen to supply energy [37]. Fatty acid levels in plasma have been found to increase after exercise, and in the current case, possibly, this is reflected by the rapid fall in urinary pantothenic acid levels post-exercise. Furthermore, the urinary levels of the adrenaline metabolite, metanephrine, are elevated and do not decline to pre-exercise levels until nearly 24 h later.

### 4.3. Microbiome Metabolites

An unexpected and wide ranging effect of exercise is on the levels of microbial metabolites in urine. Five metabolites clearly associated with the gut microflora are moderately to highly elevated by exercise and include metabolites of tryptophan, such as the uremic toxin, indoxyl sulphate, indole pyruvate and hydroxyindole pyruvate [38], the tyrosine metabolites cresol sulphate and phenol sulphate [39], as well as the histamine, metabolite urocanic acid. Elevated levels of a number of other amino acid oxidation products are observed, and these may also result from microbial activity [40]. This could have important implications for physiological function during exercise when one considers that cresol sulphate and indoxyl sulphate are potent uremic toxins. Again, this effect has been previously observed by Lustgarten *et al.*, highlighting increases in a range of products of gut microflora, including phenol sulphate, p-cresol sulphate, urocanic acid and 3-indoxyl sulphate in physically-impaired adults following an exercise programme [41]. 

### 4.4. Amino Acid Oxidation

As shown in Table 2, there are many oxidation products of amino acids produced in response to exercise that are supported in previous studies. Lustgarten *et al.* [29] observed increases in α-hydroxyisocaproate, indolelactate hydroxyisovalerate and 2-hydroxy-3-methylvalerate in their study of exercise in physically-impaired adults. They linked increases in these metabolites with PPAR-α activation. In our study, we have observed the keto acids to a greater extent than the corresponding hydroxyl acids, which is more in line with Pechlivanis *et al.*, who observed increased oxidation of branch chain fatty acids in the urine of moderately trained males post-exercise (three sets of 80-m maximal runs separated by either 10 s or 1 min) [17].

### 4.5. Carnitines 

Another group of metabolites that are affected by exercise are fatty acid carnitine conjugates. In particular, we observed increases in C12:0, C10:0 and C8:0 carnitines, which have been reported previously [42]. Romijn *et al.* [43] showed that glucose inhibited the metabolism of palmitic acid during exercise. Glycolysis is increased during exercise since two molecules of ATP are generated during the conversion of glucose to pyruvate without the requirement of oxygen. Pyruvate then enters mitochondria and is converted to acetyl CoA, which produces one molecule of NADH, but this does not require investment of ATP. In contrast, coupling of a fatty acid to CoA requires one molecule of ATP and the FADH and NADH produced during a fatty acid oxidation step. This requires oxygen in order for them to be converted to ATP in the terminal respiratory chain. Thus, it would seem logical that further oxidation of pyruvate via the Krebs cycle might take precedence over fatty acid oxidation when ATP is at a premium. In order to enter mitochondria, fatty acids have to be converted into acyl carnitines, where they are then conjugated to CoA and undergo oxidation. Carnitine conjugation is also used to transport fatty acids out of mitochondria in order to ensure that sufficient levels of free CoA are maintained; this allows the Krebs cycle to function [44]. CoA is released once acetyl CoA enters the Krebs cycle, but is required once more in the formation of succinyl CoA. Thus, in order to maintain free CoA, fatty acids conjugated to CoA may be converted to acyl carnitines and be removed from mitochondria and, ultimately, excreted. Measurement of urinary acyl carnitine levels is used to diagnose in born errors of fatty acid metabolism, which result from a defect in one of the beta oxidation steps during fatty acid metabolism in the mitochondria. In such conditions, in order to preserve free levels of CoA, the partly metabolised fatty acid is removed and excreted as its carnitine conjugate. The elevation in certain acyl carnitines may reflect something similar where fatty acids are removed to ensure the functioning of glycolysis followed by conversion of pyruvate to acetyl CoA and the oxidation of acetate in the Krebs cycle. 

### 4.6. Steroid Metabolism

The final major group of metabolites varying between the first post-exercise sample and the other samples in the series are urinary steroid metabolites. Urocortisol glucuronide, which is the major metabolite of hydrocortisone, was greatly elevated in the first post-exercise sample. Cortisol concentration is well known to vary in the blood and its metabolites, urocortisone- 3-glucuronide and urocortisol-3-glucuronide, are downstream metabolites of cortisone and cortisol. The peak times for cortisol, cortisone and urocortisone-3-glucronide concentration were found in a study of plasma levels to be in the afternoon [45]. This fits with our current observations in urine. However, from the data in Table 2, it can be seen that on the non-exercise day, the peak level was in the morning (only 50% of the peak level in the first post-exercise sample), and the afternoon samples were still lower. Thus, it would appear that exercise does influence the level of hydrocortisone metabolites. This is perhaps not surprising, since hydrocortisone is involved in many physiological functions relating to energy consumption. For instance, it has been found that corticosteroids can directly promote nitric oxide production [46]. There have been previous reports of cortisol metabolism being changed by exercise [47,48]. In addition to hydrocortisone metabolism, testosterone metabolism was also affected by exercise, and three androgen metabolites were elevated in the first post-exercise sample. There are numerous reports that testosterone is elevated in males following exercise [49]. 

## 5. Conclusions

There have been longitudinal studies looking at the collection of individual urine samples over time in normal subjects following exercise. By collecting 24-h pooled urine samples, a lot of interesting information could be lost. The metabolic changes we observed in the current pilot study have been observed across several papers, although they have not been reported in one single study [5,21,22,23,24,25,26,27,28,29,41,42,47,48,49,50,51]. It is vital, given the profound effects of exercise, that future metabolomics-based investigations also take into consideration the impact exercise has on the metabolome. For instance comparing a relatively sedentary patient cohort with a more active control group would highlight the metabolite changes due to exercise rather than changes due to disease. Another pertinent example is the carnitines, which are widely reported as disease markers [52,53]; however, given their response to exercise, differences could easily result from two groups that are not adequately matched for physical activity. Similarly, purines have been proposed as markers for various types of cancer [54], and it is obvious that normalising against creatinine would not compensate for the large fluctuations in this pathway due to physical activity. It is evident from the current study that creatinine may fluctuate over time with a similar pattern to numerous metabolites; however, it does not necessarily do so to the same degree. We therefore recommend that the most robust technique for normalisation would be to calculate subject-specific area percentages. This technique does not seem to remove any of the abundant features across the subjects, but merely acts to re-stabilise an already variant dataset, the latter considered as a norm in metabolomics investigations. In addition, it does not introduce large numbers of significant metabolites, but focuses the metabolites affected by exercise into distinct pathways, which have all been described before in various papers, ultimately adding increased weight to the observations. 

The data obtained from this simple study are extensive, and it would be possible to carry out further analysis to uncover further insights into human metabolic fluctuations. However, this will be better done on a larger sample size with better control over the dose of exercise administered. The purpose of the current study was to demonstrate the underappreciated impact of exercise when carrying out metabolomics studies and that continuous collection of all samples from individuals enables us to gain some confidence in normalising each metabolite to its total output. The current study, in fact, arose out of the difficulties we experienced when trying to collaborate in a study investigating the effects of recombinant human erythropoietin in the spot urine and plasma samples of endurance trained males taken over 10 weeks [55]. Although we expected to see an impact on the metabolome, the metabolite data were thoroughly stochastic. There was no particular information in the literature that we could turn to on this, since few studies have been conducted over such a long period. Thus, we hypothesised that, since the individuals concerned were pursuing individual training regimens, the large fluctuations in the metabolites we were observing must be due to exercise masking any other effects. Therefore, the current study supports this view and also could explain why in metabolomics studies of humans it is often difficult to find differences in cohort comparisons, since the physical activity level for an individual is not something that is often normalised. It might be possible to normalise for this using a metabolite, such as hypoxanthine or a combination of the purine markers, that is greatly affected by exercise, but this remains to be studied. The effects of exercise on the metabolome have been recently reviewed [56].

## Supplementary Materials

Supplementary materials can be accessed at: www.mdpi.com/2218-1989/5/1/119/s1.
