# Supplementary material for: A Study of the Effects of Exercise on the Urinary Metabolome Using Normalisation to Individual Metabolic Output
Source: Metabolites. 2015 Feb 27;5(1):119–39. doi: 10.3390/metabo5010119 (PMC4381293; doi:10.3390/metabo5010119)
Supplement: Supplementary file 1 [file metabolites-05-00119-s001.pdf]

Supplementary Materials

MzMatch Processing Settings with Script

library(rJava)
library(mzmatch.R)
mzmatch.init()

```
setwd()
mzXMLfiles <- dir(full.names=TRUE,pattern="\\.mzXML$",recursive=TRUE)
outputfiles <- paste(sub(".mzXML","",mzXMLfiles),".peakml",sep="")


xset <- xcmsSet(mzXMLfiles, method='centWave', ppm=2, peakwidth=c(5,100), snthresh=3, prefilter=c(3,1000), integrate=1, mzdiff=0.001, verbose.columns=TRUE, fitgauss=FALSE, nSlaves=16)

## Afterwards you split resulting xset by filenames and process it with peakml file writer.

xsets <- split (xset,xset@filepaths)

peakMLparallel <- function(x)
{
library(mzmatch.R)
mzmatch.init ()
xset <- xsets[[x]]
PeakML.xcms.write.SingleMeasurement
(xset=xset,filename=outputfiles[x],ionisation="detect",addscans=20,writeRejected=FALSE,ApodisationFilter=TRUE)
}


if (length(xsets)==length(mzXMLfiles))
{

## Create a snow cluster for writing peakml files
cl <- makeCluster (8, type="SOCK")
clusterExport (cl,list=c("xsets","outputfiles"))
system.time(clusterApply(cl,1:length(outputfiles),peakMLparallel))
stopCluster(cl)
} else
{
cat ("xcms set does not contains peaks for all mzXML samples.")
}


MainClasses <- dir ()
dir.create ("combined_RSD_filtered")
dir.create ("combined_RSD_rejected")
dir.create ("combined")
for (i in 1:length(MainClasses)){FILESf <- dir (MainClasses[i],full.names=TRUE,pattern="\\.peakml$",recursive=TRUE)
OUTPUTf <- paste ("combined/",MainClasses[i],".peakml",sep="")
if(length(FILESf)>0){mzmatch.ipeak.Combine
(i=paste(FILESf,collapse=","),v=T,rtwindow=30,o=OUTPUTf,combination="set",ppm=5,label=paste
(MainClasses[i],sep=""))
RSDf <- paste ("combined_RSD_filtered/",MainClasses[i],".peakml",sep="")
REJf <- paste ("combined_RSD_rejected/",MainClasses[i],".peakml",sep="")
if(length(FILESf)>1)
mzmatch.ipeak.filter.RSDFilter(i=OUTPUTf,o=RSDf,rejected=REJf,rsd=1,v=T) else file.copy(OUTPUTf,RSDf)}}
INPUTDIR <- "combined_RSD_filtered"
FILESf <- dir (INPUTDIR,full.names=TRUE,pattern="\\.peakml$")
mzmatch.ipeak.Combine(i=paste(FILESf,collapse=","),v=T,rtwindow=30,
o="combined.peakml",combination="set",ppm=5)
mzmatch.ipeak.filter.NoiseFilter
(i="combined.peakml",o="combined_noisef.peakml",v=T,codadw=0.8)
mzmatch.ipeak.filter.SimpleFilter(i="combined_noisef.peakml", o="combined_sfdet.peakml", mindetections=3, v=T)
```

```
mzmatch.ipeak.filter.SimpleFilter(i="combined_sfdet.peakml",
o="combined_highintensity.peakml", minintensity=1000, v=T)
PeakML.GapFiller(filename = "combined_highintensity.peakml", ionisation = "detect", Rawpath
= NULL, outputfile = "highintensity_gapfilled.peakml", ppm = 0, rtwin = 0)
mzmatch.ipeak.sort.RelatedPeaks  (JHeapSize=5000,  i="highintensity_gapfilled.peakml",  v=T,
o="mzMatch_output.peakml",basepeaks="mzMatch_basepeaks.peakml",ppm=5,rtwindow=6)
annot <- paste("relation.id,relation.ship,codadw,charge")
mzmatch.ipeak.convert.ConvertToText       (JHeapSize=5000,       i="mzMatch_output.peakml",
o="mzMATCHoutput.txt",v=T,annotations=annot)

# Processing finished!     Now extracting chromatogram images for each peak.
chromdir <- "chromatograms"
dir.create(chromdir)
PeakMLData <- PeakML.Read("mzMatch_output.peakml",Rawpath=NULL)
peakIDlist <- c(1:length(unique(PeakMLData$peakDataMtx[,10])))
sampnames <- PeakMLData$sampleNames
sampleslist<-c(1:max(PeakMLData$peakDataMtx[,9]))
groupsets <- max(PeakMLData$peakDataMtx[,11])
if (groupsets!=1) {samplegroups <- PeakMLData$phenoData} else {samplegroups <- sampnames}
classnumbers <- samplegroups
for  (i  in  1:length(unique(samplegroups))){classnumbers  <-  sub(unique(classnumbers)[i],  i,
classnumbers)}
for (a in 1:length(peakIDlist)){peakID <- peakIDlist[a]
hits <- which(PeakMLData$peakDataMtx[,10]==peakID)
intslist <- vector ("list")
rtlist <- vector ("list")
for (i in 1:length(hits)){intslist[[i]] <- PeakMLData$chromDataList[[hits[i]]][2,]
rtlist[[i]] <- (PeakMLData$chromDataList[[hits[i]]][3,]) / 60 }
maxint <- max(unlist(intslist))
minrt <-  min (unlist(rtlist))
maxrt <- max (unlist(rtlist))
samplenumbers <- PeakMLData$peakDataMtx[hits,9]
myfilename <- paste(getwd(),"/",chromdir,"/",peakID,".png",sep="")
png(myfilename, width = 350, height = 300)
lw = 0.2
par(fig=c(0,1-lw,0,1))
plot (1,1,xlab="RT (m)", ylab="Intensity", pch="", xlim=c(minrt,maxrt), ylim=c(0,maxint))
for (i in 1:length(hits)){if (PeakMLData$peakDataMtx[hits[i],9] %in% sampleslist==TRUE){
points (rtlist[[i]], intslist[[i]], type="l", col=classnumbers[samplenumbers [i]])}}
par(fig=c(0,1,0,1))
lpos <- par("usr")[2]-(lw /(1-lw ))*(par("usr")[2]-par("usr")[1])
legend       (lpos,       par("usr")[4],       text.col=unique(classnumbers[sampleslist]),
unique(samplegroups[sampleslist]), cex=0.8,xpd=TRUE)
dev.off()}


## Rename file based on folder name
RENAME<-paste(basename(getwd()),".txt",sep="")
file.rename("mzMATCHoutput.txt",RENAME)

## Rename file based on folder name
RENAME<-paste(basename(getwd()),".peakml",sep="")
file.rename("mzMatch_output.peakml",RENAME)

### folder renaming
OLDNAME<-getwd()
RENAME<-paste(getwd(),"-done",sep="")
setwd()
file.rename(OLDNAME,RENAME)
```

Table S1. Ideom settings.

Category	Parameter	Input
XCMS (Centwave)	Method (file type)	mzXML
	Ppm	2
	Peak width (min)	5 s
	Peak width (max)	100 s
	S/N * threshold	3
	Pre-filter (No. of points)	3
	Pre-filter (intensity)	1000
	m/*z* difference	0.001
MzMatch	MzMatch grouping RT ~ window	0.5 min
	MzMatch grouping *m*/*z* ppm	5 ppm
	Relative SD (RSD) filter	0.80 #
	Noise filter (CODA-DW ¥)	0.80
	Intensity filter (LOQ α)	1000
	Minimum detections #	3
	RT window for related peaks	0.10 min
Ideom	RT for id of authentic standards	5.0%
	RT for id for calculated RT	50.0%
	Ppm for mass identification	3.0 ppm
	Ignore related peaks before RT	0.0 min
	RT window for complex adducts	0.50 min
	RT window for duplicate peaks	1.00 min
	RT window for shoulder peaks	2.0 min
	Intensity ratio for shoulder peaks	5 to 1
	Intensity limit duplicate peaks	1%
	r^2 limit for duplicate peaks	0.99
	Preferred database	HumanDB (has β)

Input details for settings in Ideom version 19 excel file. * Signal to noise; ~ retention time; # generous; ¥ Durbin–Watson criterion; α limit of quantification; β Homo sapiens.

© 2015 by the authors; licensee MDPI, Basel, Switzerland. This article is an open access article distributed under the terms and conditions of the Creative Commons Attribution license (http://creativecommons.org/licenses/by/4.0/).
