# Supplementary material for: A Study of the Effects of Exercise on the Urinary Metabolome Using Normalisation to Individual Metabolic Output
Source: Metabolites. 2015 Feb 27;5(1):119–39. doi: 10.3390/metabo5010119 (PMC4381293; doi:10.3390/metabo5010119)
Supplement: Supplementary file 2 [file metabolites-05-00119-s002.docx]

Supplementary Materials

MzMatch Processing Settings with Script

library(rJava)

library(mzmatch.R)

mzmatch.init()

setwd()

mzXMLfiles <- dir(full.names=TRUE,pattern="\\.mzXML$",recursive=TRUE)

outputfiles <- paste(sub(".mzXML","",mzXMLfiles),".peakml",sep="")

xset <- xcmsSet(mzXMLfiles, method='centWave', ppm=2, peakwidth=c(5,100), snthresh=3, prefilter=c(3,1000), integrate=1, mzdiff=0.001, verbose.columns=TRUE,

fitgauss=FALSE, nSlaves=16)

## Afterwards you split resulting xset by filenames and process it with peakml file writer.

xsets <- split (xset,xset@filepaths)

peakMLparallel <- function(x)

{

library(mzmatch.R)

mzmatch.init ()

xset <- xsets[[x]]

PeakML.xcms.write.SingleMeasurement (xset=xset,filename=outputfiles[x],ionisation="detect",addscans=20,writeRejected=FALSE,ApodisationFilter=TRUE)

}

if (length(xsets)==length(mzXMLfiles))

{

## Create a snow cluster for writing peakml files

cl <- makeCluster (8, type="SOCK")

clusterExport (cl,list=c("xsets","outputfiles"))

system.time(clusterApply(cl,1:length(outputfiles),peakMLparallel))

stopCluster(cl)

} else

{

cat ("xcms set does not contains peaks for all mzXML samples.")

}

MainClasses <- dir ()

dir.create ("combined_RSD_filtered")

dir.create ("combined_RSD_rejected")

dir.create ("combined")

for (i in 1:length(MainClasses)){FILESf <- dir (MainClasses[i],full.names=TRUE,pattern="\\.peakml$",recursive=TRUE)

OUTPUTf <- paste ("combined/",MainClasses[i],".peakml",sep="")

if(length(FILESf)>0){mzmatch.ipeak.Combine (i=paste(FILESf,collapse=","),v=T,rtwindow=30,o=OUTPUTf,combination="set",ppm=5,label=paste(MainClasses[i],sep=""))

RSDf <- paste ("combined_RSD_filtered/",MainClasses[i],".peakml",sep="")

REJf <- paste ("combined_RSD_rejected/",MainClasses[i],".peakml",sep="")

if(length(FILESf)>1) mzmatch.ipeak.filter.RSDFilter(i=OUTPUTf,o=RSDf,rejected=REJf,rsd=1,v=T) else file.copy(OUTPUTf,RSDf)}}

INPUTDIR <- "combined_RSD_filtered"

FILESf <- dir (INPUTDIR,full.names=TRUE,pattern="\\.peakml$")

mzmatch.ipeak.Combine(i=paste(FILESf,collapse=","),v=T,rtwindow=30, o="combined.peakml",combination="set",ppm=5)

mzmatch.ipeak.filter.NoiseFilter (i="combined.peakml",o="combined_noisef.peakml",v=T,codadw=0.8)

mzmatch.ipeak.filter.SimpleFilter(i="combined_noisef.peakml", o="combined_sfdet.peakml", mindetections=3, v=T)

mzmatch.ipeak.filter.SimpleFilter(i="combined_sfdet.peakml", o="combined_highintensity.peakml", minintensity=1000, v=T)

PeakML.GapFiller(filename = "combined_highintensity.peakml", ionisation = "detect", Rawpath = NULL, outputfile = "highintensity_gapfilled.peakml", ppm = 0, rtwin = 0)

mzmatch.ipeak.sort.RelatedPeaks (JHeapSize=5000, i="highintensity_gapfilled.peakml", v=T, o="mzMatch_output.peakml",basepeaks="mzMatch_basepeaks.peakml",ppm=5,rtwindow=6)

annot <- paste("relation.id,relation.ship,codadw,charge")

mzmatch.ipeak.convert.ConvertToText (JHeapSize=5000, i="mzMatch_output.peakml", o="mzMATCHoutput.txt",v=T,annotations=annot)

# Processing finished! Now extracting chromatogram images for each peak.

chromdir <- "chromatograms"

dir.create(chromdir)

PeakMLData <- PeakML.Read("mzMatch_output.peakml",Rawpath=NULL)

peakIDlist <- c(1:length(unique(PeakMLData$peakDataMtx[,10])))

sampnames <- PeakMLData$sampleNames

sampleslist<-c(1:max(PeakMLData$peakDataMtx[,9]))

groupsets <- max(PeakMLData$peakDataMtx[,11])

if (groupsets!=1) {samplegroups <- PeakMLData$phenoData} else {samplegroups <- sampnames}

classnumbers <- samplegroups

for (i in 1:length(unique(samplegroups))){classnumbers <- sub(unique(classnumbers)[i], i, classnumbers)}

for (a in 1:length(peakIDlist)){peakID <- peakIDlist[a]

hits <- which(PeakMLData$peakDataMtx[,10]==peakID)

intslist <- vector ("list")

rtlist <- vector ("list")

for (i in 1:length(hits)){intslist[[i]] <- PeakMLData$chromDataList[[hits[i]]][2,]

rtlist[[i]] <- (PeakMLData$chromDataList[[hits[i]]][3,]) / 60 }

maxint <- max(unlist(intslist))

minrt <- min (unlist(rtlist))

maxrt <- max (unlist(rtlist))

samplenumbers <- PeakMLData$peakDataMtx[hits,9]

myfilename <- paste(getwd(),"/",chromdir,"/",peakID,".png",sep="")

png(myfilename, width = 350, height = 300)

lw = 0.2

par(fig=c(0,1-lw,0,1))

plot (1,1,xlab="RT (m)", ylab="Intensity", pch="", xlim=c(minrt,maxrt), ylim=c(0,maxint))

for (i in 1:length(hits)){if (PeakMLData$peakDataMtx[hits[i],9] %in% sampleslist==TRUE){

points (rtlist[[i]], intslist[[i]], type="l", col=classnumbers[samplenumbers [i]])}}

par(fig=c(0,1,0,1))

lpos <- par("usr")[2]-(lw /(1-lw ))*(par("usr")[2]-par("usr")[1])

legend (lpos, par("usr")[4], text.col=unique(classnumbers[sampleslist]), unique(samplegroups[sampleslist]), cex=0.8,xpd=TRUE)

dev.off()}

## Rename file based on folder name

RENAME<-paste(basename(getwd()),".txt",sep="")

file.rename("mzMATCHoutput.txt",RENAME)

## Rename file based on folder name

RENAME<-paste(basename(getwd()),".peakml",sep="")

file.rename("mzMatch_output.peakml",RENAME)

### folder renaming

OLDNAME<-getwd()

RENAME<-paste(getwd(),"-done",sep="")

setwd()

file.rename(OLDNAME,RENAME)

**Table S1.** Ideom settings.

| **Category** | **Parameter** | **Input** |
| --- | --- | --- |
| XCMS (Centwave) | Method (file type) | mzXML |
|  | Ppm | 2 |
|  | Peak width (min) | 5 s |
|  | Peak width (max) | 100 s |
|  | S/N ^*^ threshold | 3 |
|  | Pre-filter (No. of points) | 3 |
|  | Pre-filter (intensity) | 1000 |
|  | *m*/*z* difference | 0.001 |
| MzMatch | MzMatch grouping RT ^~^ window | 0.5 min |
|  | MzMatch grouping *m*/*z* ppm | 5 ppm |
|  | Relative SD (RSD) filter | 0.80 ^#^ |
|  | Noise filter (CODA-DW ^¥^) | 0.80 |
|  | Intensity filter (LOQ ^α^) | 1000 |
|  | Minimum detections ^#^ | 3 |
|  | RT window for related peaks | 0.10 min |
| Ideom | RT for id of authentic standards | 5.0% |
|  | RT for id for calculated RT | 50.0% |
|  | Ppm for mass identification | 3.0 ppm |
|  | Ignore related peaks before RT | 0.0 min |
|  | RT window for complex adducts | 0.50 min |
|  | RT window for duplicate peaks | 1.00 min |
|  | RT window for shoulder peaks | 2.0 min |
|  | Intensity ratio for shoulder peaks | 5 to 1 |
|  | Intensity limit duplicate peaks | 1% |
|  | r^2^ limit for duplicate peaks | 0.99 |
|  | Preferred database | HumanDB (has ^β^) |

Input details for settings in Ideom version 19 excel file. * Signal to noise; ~ retention time; ^#^ generous;
^¥^ Durbin–Watson criterion; ^α^ limit of quantification; ^β^ Homo sapiens.

© 2015 by the authors; licensee MDPI, Basel, Switzerland. This article is an open access article distributed under the terms and conditions of the Creative Commons Attribution license (http://creativecommons.org/licenses/by/4.0/).
